# Virtual reality volumetric rendering versus cross-sectional imaging for pancreatic cancer resectability assessment: a pilot randomized controlled reader study

**DOI:** 10.1186/s42234-026-00202-2

**Published:** 2026-03-09

**Authors:** Karl Eisenträger, Kaya Saribeyoglu, Uli Fehrenbach, Matthäus Felsenstein, Lea Timmermann, Pedro Lua Machado Pereira, Wenzel Schöning, Benjamin Strücker, Johann Pratschke, Andreas Pascher, Thomas Malinka, Igor Maximilian Sauer, Haluk Morgul, Moritz Queisner

**Affiliations:** 1 Department of Surgery, Experimental Surgery, CCM|CVK Charité – Universitätsmedizin Berlin, corporate member of Freie Universität Berlin and Humboldt-Universität Zu Berlin, Berlin, Germany; 2 Department of Radiology, Charité – Universitätsmedizin Berlin, corporate member of Freie Universität Berlin and Humboldt-Universität Zu Berlin, Berlin, Germany; 3 Cluster of Excellence Matters of Activity, Funded By the Deutsche Forschungsgemeinschaft (DFG, German Research Foundation) Under Germany’s Excellence Strategy - EXC 2025 - 390648296, Image Space Material, Berlin, Germany; 4 Department of General, Visceral and Transplant Surgery, University Hospital Münster, Münster, Germany

**Keywords:** Virtual Reality, Cancer, Resectability, Inter-Rater Agreement, Volumetric Rendering, Surgery Planning

## Abstract

**Background:**

Current imaging assessment for pancreatic cancer resectability demonstrates problematic inter-observer variability, with only fair-to-moderate agreement among experienced raters. Virtual reality technology offers stereoscopic three-dimensional visualization that may improve diagnostic accuracy and agreement. However, optimal visualization strategies for clinical adoption remain unclear.

**Methods:**

Ten hepatopancreatobiliary surgeons from two high-volume centers were randomized 1:1 to assess twelve contrast-enhanced CT cases using either VR volumetric rendering or CSI. Primary outcomes included inter-rater agreement, diagnostic accuracy against expert reference standard, assessment time, and surgeon confidence. Statistical analysis employed Fleiss’ κ for inter-rater agreement and two-sided Mann–Whitney U tests on surgeon-level summary measures for between-group comparisons.

**Results:**

CSI display on 2D screens achieved substantial inter-rater agreement for resectability assessment (κ = 0.609) while VR demonstrated only slight agreement (κ = 0.127). Diagnostic accuracy was superior with CSI (84.7% vs. 79.7%), with the most pronounced difference in resectability determination (83.3% vs. 58.3%, *p* = 0.033). VR users reported significantly lower confidence (4.85 ± 1.15 vs. 6.32 ± 0.77, *p* = 0.028). Assessment times were comparable between groups (median 313.5 s vs. 327.5 s, *p* = 1.00).

**Conclusions:**

In this preliminary investigation, our VR visualization strategy demonstrated lower diagnostic accuracy and inter-rater agreement than CSI. However, prior studies suggest that VR systems employing alternative, hybrid visualization approaches may improve inter-rater agreement, indicating that visualization strategy, rather than VR technology per se, is the primary determinant of utility.

**Trial registration:**

DRKS00033932 (German Clinical Trials Register), registered prospectively.

**Supplementary Information:**

The online version contains supplementary material available at 10.1186/s42234-026-00202-2.

## Background

Pancreatic ductal adenocarcinoma (PDAC) remains one of oncology’s greatest challenges, ranking as the seventh leading cause of cancer mortality globally (Rawla et al., 2019) and third in the United States and Europe (Ilic and Ilic 2022). With five-year survival rates of only 5–13% and population-level resection rates averaging just 14%, accurate resectability assessment is critical for optimizing patient selection for potentially curative surgery (Lockie et al., 2025). Late-stage presentation remains a fundamental barrier to curative treatment, as most patients are diagnosed beyond the window of resectable disease; however, emerging serum metabolite biomarkers have demonstrated promising accuracy for Stage-I detection (Cao et al., 2021). 10–36% of patients radiologically deemed resectable ultimately do not undergo resection, underscoring fundamental limitations in current imaging-based assessment methods (Pecorelli et al., 2022).

The National Comprehensive Cancer Network (NCCN) guidelines classify PDAC resectability based on tumor-vessel relationships visualized on imaging (Updates 2024). Despite standardized criteria, inter-observer agreement for resectability assessment remains only fair to moderate using conventional imaging (Badgery et al., 2023; Giannone et al., 2021), with one-third of cases receiving all three resectability classifications across experienced raters (Giannone et al., 2021). This problematic variability in anatomical assessment prompted our exploration of virtual reality (VR) volumetric rendering as a three-dimensional visualization modality to potentially improve accuracy and agreement among decision makers.

Wearing a head-mounted display, physicians can assess medical imaging data as a stereoscopic 3D model instead of a 2D image sequence. Additionally, VR visualizations enable manipulation of resection planes from any perspective and modulation of tissue opacity to fade surrounding structures. Recent regulatory developments have accelerated VR’s clinical integration: multiple VR DICOM viewers have received FDA 510(k) clearance and European CE marking (Home n.d.; Disclaimers 2023). As VR medical software certification becomes increasingly common, understanding optimal visualization strategies is critical for clinical adoption.

This randomized, controlled reader study compares VR volumetric renderings with cross-sectional imaging (CSI; 2D slice display) for PDAC resectability assessment among specialized pancreatic surgeons, focusing on inter-rater agreement, diagnostic accuracy, efficacy, and surgeon confidence.

## Methods

This study was conducted in accordance with the Declaration of Helsinki at Charité – Universitätsmedizin Berlin and Universitätsklinik Münster. Ethical approval was granted by respective ethics committees (Ethikkommission der Charité—Universitätsmedizin Berlin EA1/129/24; Ethikkommission Westfalen-Lippe 2024–852-b-S). The trial was registered in the German Clinical Trials Register (DRKS00033932). All participating medical professionals provided written informed consent. Patient data was included only where individuals had previously authorized general research use through Charité – Universitätsmedizin Berlin's institutional consent process. The authors used Claude Sonnet 4.5 (Anthropic) to assist in drafting and refining portions of the manuscript.

### Participants

Eligibility was limited to fully trained hepatobiliary and pancreatic surgeons actively practicing at specialized pancreatic cancer centers performing over 75 pancreas surgeries annually. All study procedures were explained, and participants provided written informed consent before enrollment. Ten surgeons from two surgical departments participated in this study.

### Study procedure

Participants were randomized 1:1 to VR volumetric rendering or CSI using stratified minimization by surgical experience level (intermediate, advanced) (Joo et al., 2019). The allocation algorithm employed weighted scoring (40% within-stratum, 60% overall balance) with random tie-breaking. Randomization sequences were computer-generated, and allocation concealment was maintained until the point of assignment.

Prior to case assessment, participants provided baseline information including visual acuity confirmation, age, years of experience as a surgeon, number of pancreatic resections performed, and prior VR experience for medical data visualization. Each participant was introduced to the use of the respective tool with an example case. Training consisted of a standardized orientation using a single example case; no timed training period, competency assessment, or practice set of study cases was performed prior to evaluation.

An expert pancreatic radiologist who regularly participates in multidisciplinary tumor boards independently evaluated all cases using CSI and the identical questionnaire, providing the reference standard for accuracy comparisons across both modalities.

Each participant evaluated 12 pancreatic tumor cases sequentially with automated timestamp recording at each decision point. For each case, participants completed anatomical evaluation including arterial variations, stenosis (celiac trunk and superior mesenteric artery), and tumor location within pancreatic segments. Participants then identified arterial tumor contact (aorta, superior mesenteric artery, splenic artery, celiac trunk, common hepatic artery, proper hepatic artery, gastroduodenal artery, arterial variations, or none) and specified contact degree (< 180° or > 180°). Venous involvement was similarly assessed (portal vein, superior mesenteric vein, other veins, or none) with corresponding degree specifications. Following vascular assessment, participants classified tumor resectability (resectable, borderline-resectable, or locally advanced per NCCN Guidelines) and rated confidence on a 7-point Likert scale (1:”very unsure” to 7:”very sure”).

### VR and CSI equipment

VR volumetric rendering was performed using *Medical Holodeck* (Medical Holodeck AG, Zurich, Switzerland) for volumetric CT rendering with transfer function set to −125 to 225 Hounsfield Units with slight transparency to approximate CSI abdominal windowing (Medical n.d.). Participants could rotate, resize, and apply cutting planes but were not trained in transfer function adjustment due to time constraints and software novelty. Hardware comprised a Windows PC (64 GB RAM, Geforce RTX 3090 with 24 GB of GDDR6X memory, NVIDIA Corporation, Santa Clara, California), Quest 3 headset (Meta Platforms Inc., Menlo Park, USA) connected via Air-Link through a TP-Link Archer AXE75 AX5400 Wi-Fi 6E router (TP-Link Technologies Co., Ltd., Shenzhen, China) set up as an access point. The CSI group used a MacBook Pro M4 (Apple Inc., Cupertino, CA, USA) connected to a 4 K monitor and reviewed CT data as sequential 2D slices using OsiriX MD (Pixmeo SARL, Geneva, Switzerland).

### Imaging data

Twelve pancreatic tumor cases were evaluated using contrast-enhanced spiral CT with arterial and portal-venous phases acquired on modern multi-detector CT systems (0.625 mm slice thickness) to optimize visualization of pancreatic parenchyma, tumor, and vascular anatomy.

### Statistical analysis

All statistical analyses were performed using Python 3.9.0 (Python Software Foundation; pandas, NumPy, SciPy, statsmodels, Matplotlib, seaborn) (Rossum 2020).

Inter-rater agreement for categorical outcomes was quantified using Fleiss’ κ (κ). Because κ is sensitive to prevalence and marginal distributions, we additionally reported observed percent agreement (mean per-case pairwise agreement across raters, averaged across the 12 cases) and Gwet’s AC1 as complementary agreement metrics. Overall agreement was calculated across the 10 study surgeons (VR + CSI combined; expert excluded) and repeated within each visualization group separately (VR *n* = 5; CSI *n* = 5). When all raters selected the same category for all cases (no variance), κ was reported as undefined. Diagnostic accuracy was computed by comparing each surgeon’s ratings to the expert radiologist reference standard (proportion of exact matches across cases), summarized descriptively by group. Between-group comparisons for outcomes measured repeatedly per case (accuracy, time, confidence) were performed on surgeon-level summary measures (one value per surgeon) using two-sided Mann–Whitney U tests (α = 0.05). Continuous participant characteristics were compared between groups using Welch’s t-test (two-sided, α = 0.05). For total assessment time, a sensitivity analysis excluding outliers (1.5 × IQR rule) was performed and group comparisons were repeated. Correlations reported across cases were assessed using Pearson’s correlation coefficient (two-sided, α = 0.05).

## Results

### Participant characteristics

Ten experienced surgeons participated, equally randomized to VR (*n* = 5) or CSI (*n* = 5). The VR group had slightly more experience (mean: 19.6 ± 10.1 years) than the CSI group (14.0 ± 5.4 years), without statistically significance (t = 1.09, df = 8, *p* = 0.31), with similar ages (46.0 ± 9.4 vs. 45.4 ± 6.9 years). Regarding surgical volume, the VR group had 3 surgeons with 90 + pancreatic resections and 2 with 21–50 resections, while the CSI group had 4 surgeons with 90 + resections and 1 with 21–50 resections. All were board-certified surgeons with hepatopancreatobiliary subspecialty training. All VR participants had prior VR exposure but were not regular users or used VR clinically.

### Assessment times

Total assessment times were comparable between groups (VR: 313.5 s [IQR: 254.8—444.2 s] vs CSI: 327.5 s [IQR: 260.2—416.8 s], *p* = 1.00). Outlier removal did not materially change this finding (VR: 301.0 s vs. CSI: 321.0 s). Task-specific median times were similar between groups; no task-level comparison reached statistical significance when analyzed at the surgeon level (all *p* ≥ 0.24): (VR: 90.5 s vs. CSI: 86.0 s), SMA stenosis (both: 3.0 s), tumor position (VR: 41.5 s vs. CSI: 45.5 s), arterial involvement (VR: 72.0 s vs CSI: 72.5 s), venous involvement (VR: 53.0 s vs CSI: 40.5 s), or resectability assessment (VR: 5.0 s vs CSI: 4.0 s). Coeliac trunk stenosis assessment showed a descriptive difference (VR: 8.0 s vs. CSI: 20.0 s) but this did not reach statistical significance when analyzed at the surgeon level (*p* = 0.69); this 12-s reduction lacks practical significance in the context of surgical planning.

### Overall inter-rater agreement analysis

Inter-rater agreement was quantified using Fleiss’ κ (reported as κ) across 10 participants (VR + CSI; expert excluded). As complementary metrics (to address κ sensitivity to prevalence/marginals), we also report observed percent agreement and Gwet’s AC1.

Analysis across 10 participants and 12 cases revealed variable inter-rater agreement across anatomical features. Tumor position localization showed the highest reliability (κ = 0.717; 84.3% agreement), followed by vascular stenosis assessments (coeliac trunk κ = 0.638; 84.8% agreement, superior mesenteric artery κ = 0.593; 89.6% agreement). Arterial contact assessments varied widely: splenic artery (κ = 0.766; 88.5% agreement) and superior mesenteric artery (κ = 0.612; 94.1% agreement) demonstrated substantial agreement, while proper hepatic and gastroduodenal arteries showed agreement worse than chance (κ = −0.017 and κ = −0.027, respectively) despite high observed agreement for proper hepatic artery contact (96.7%), highlighting the known prevalence/marginal sensitivity of κ. Venous involvement demonstrated fair agreement (superior mesenteric vein κ = 0.336;68.1% agreement, portal vein κ = 0.265; 63.5% agreement). Overall resectability assessment achieved only fair agreement (κ = 0.360; 61.9% agreement). As a prevalence-robust sensitivity metric, Gwet’s AC1 yielded results consistent with Fleiss’ κ, with resectability showing AC1 = 0.457 (overall across 10 surgeons).

### Group-specific inter-rater agreement analysis by visualization method

**Fig. 1:**
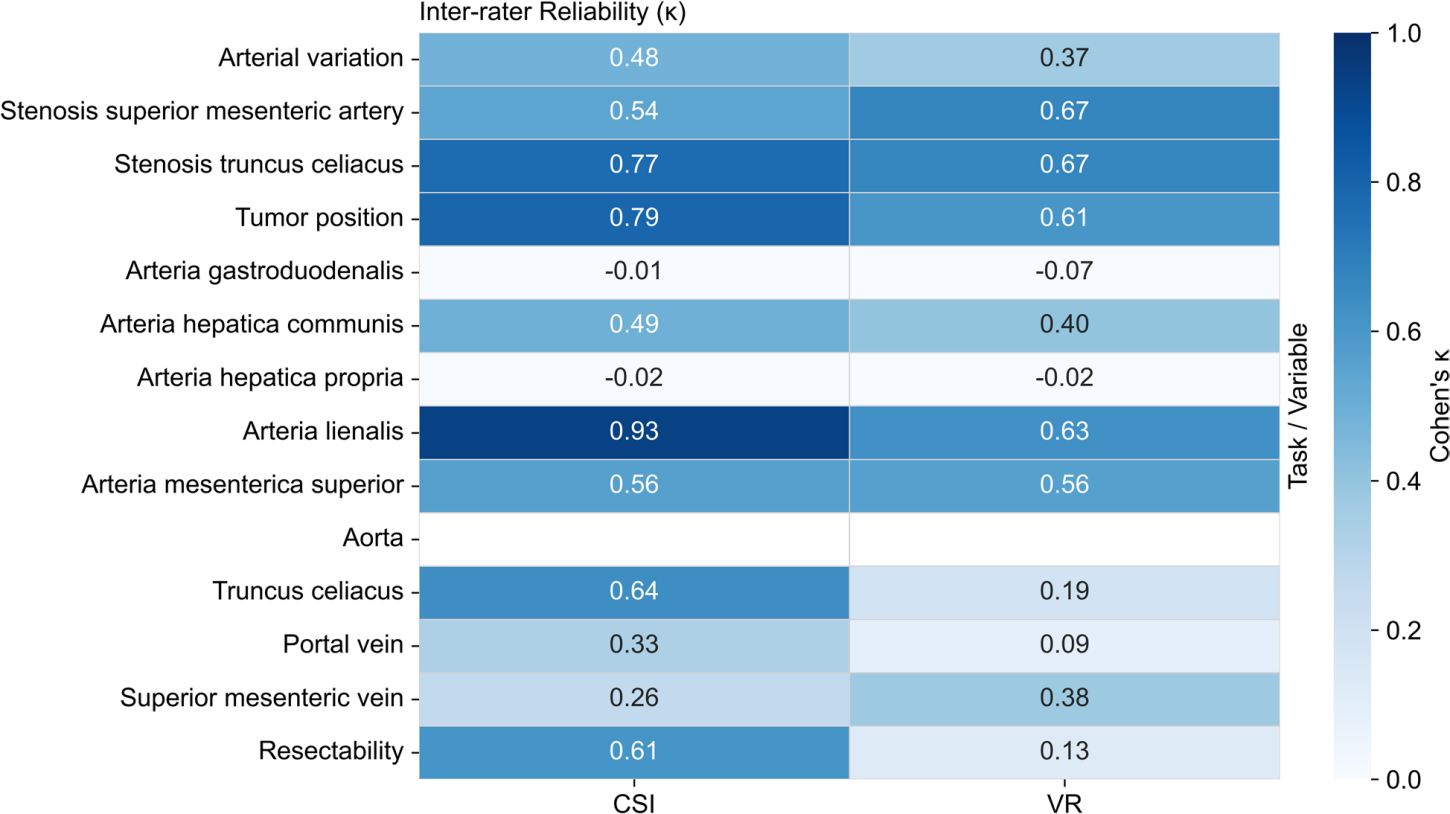
Inter-rater Reliability (κ) Comparison Between CSI and VR by Anatomical Variable. Heatmap displaying Fleiss’ κ coefficients for inter-rater agreement across 14 anatomical assessment variables. The left column shows CSI imaging results (*n* = 5 surgeons); the right column shows VR volumetric rendering results (*n* = 5 surgeons). Color intensity represents agreement strength: darker blue indicates higher agreement (κ approaching 1.0), lighter blue indicates lower agreement. White cells indicate undefined κ due to perfect consensus (all raters chose the same category)

**Fig. 2:**
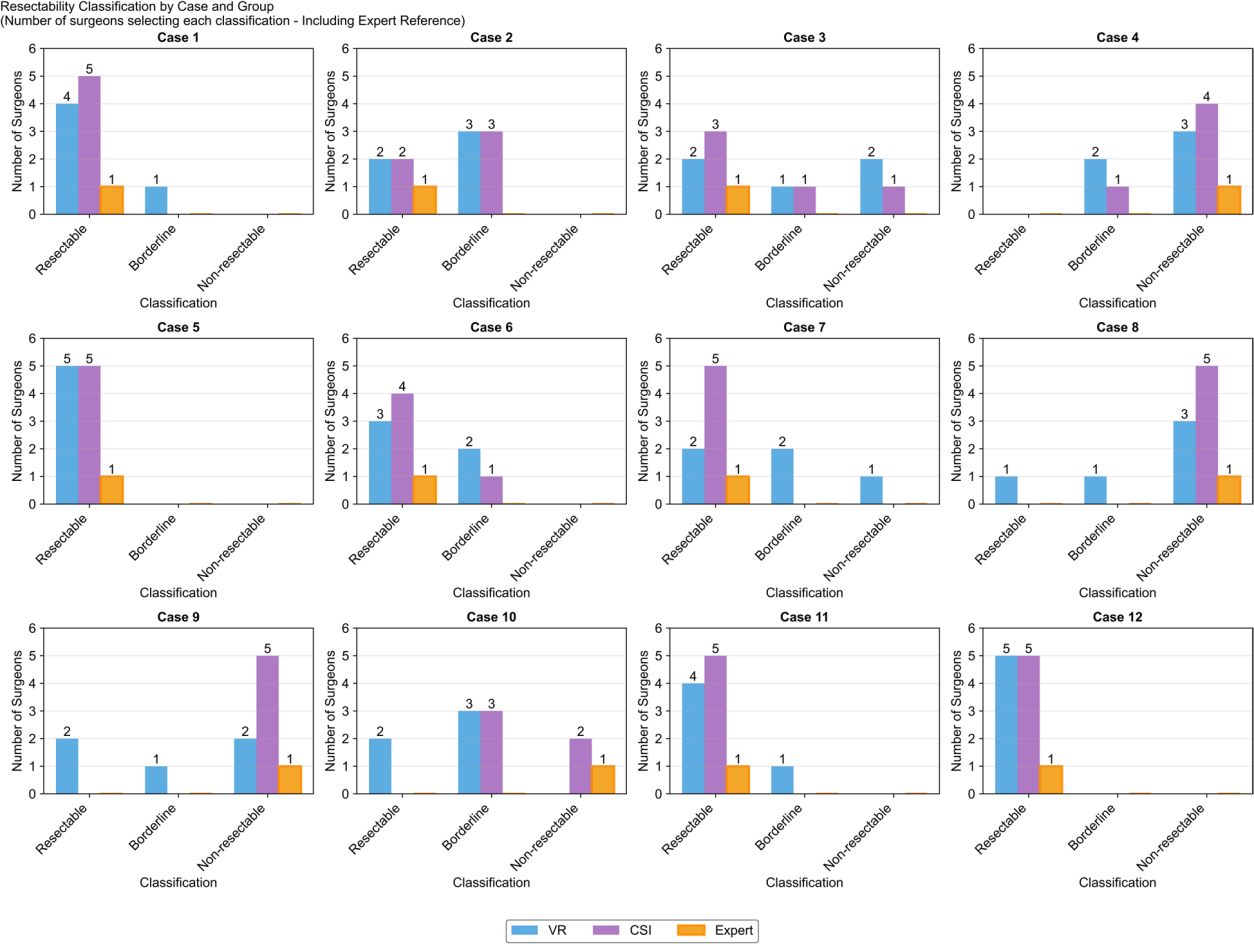
Resectability classification by case and visualization method. Distribution of resectability classifications (Resectable, Borderline, Non-resectable) for 12 pancreatic tumor cases by VR group (*n* = 5, blue), CSI group (*n* = 5, purple), and expert radiologist (orange). Y-axis shows the number of surgeons selecting each classification

CSI (*n* = 5) versus VR (*n* = 5) revealed substantial differences in inter-rater reliability across multiple parameters (Figs. 1 and 2).

For resectability assessment, CSI achieved substantial agreement (κ = 0.609) while VR demonstrated only slight agreement (κ = 0.127). Gwet’s AC1 corroborated the κ findings for resectability, showing higher agreement with CSI (AC1 = 0.684) than VR (AC1 = 0.249).Tumor position assessment showed substantial agreement in both groups, with CSI superior (CSI κ = 0.792 vs. VR κ = 0.606).

Vascular stenosis maintained substantial agreement in both modalities, with coeliac trunk stenosis showing κ = 0.769 (CSI) versus κ = 0.672 (VR), and superior mesenteric artery stenosis κ = 0.542 versus κ = 0.673 respectively.

Arterial contact assessments revealed striking disparities. Splenic artery contact achieved almost perfect agreement with CSI (κ = 0.932) versus substantial with VR (κ = 0.627). Coeliac trunk contact showed substantial agreement with CSI (κ = 0.639) but only slight with VR (κ = 0.191). Common hepatic artery demonstrated moderate agreement in both groups (CSI κ = 0.489, VR κ = 0.405).

Venous assessments showed variable patterns: portal vein contact achieved fair agreement with CSI (κ = 0.330) but only slight with VR (κ = 0.091), while superior mesenteric vein showed fair agreement in both groups (CSI κ = 0.259, VR κ = 0.377).

### Decision confidence

**Fig. 3:**
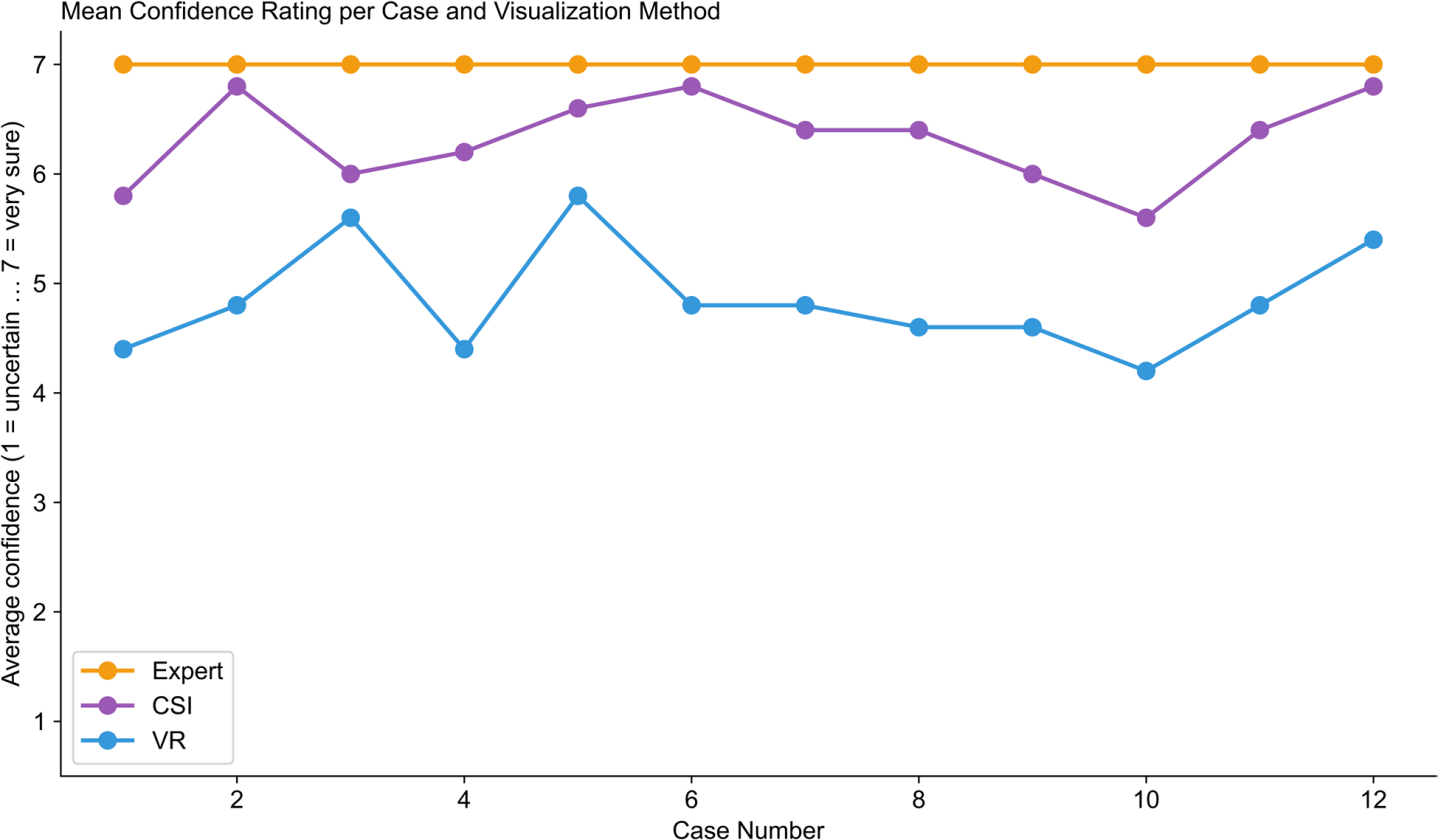
Mean confidence rating per case and visualization method. Line graph displaying average surgeon confidence scores (1 = very uncertain to 7 = very sure) across 12 pancreatic tumor cases for three groups: expert radiologist reference (orange circles), CSI group (purple circles), and VR group (blue circles)

Surgeon confidence differed significantly between visualization methods (two-sided Mann–Whitney U test on surgeon-level mean confidence across 12 cases per surgeon, *p* = 0.028). CSI users reported substantially higher confidence (mean 6.32 ± 0.77, median 6.0) compared to VR users (mean 4.85 ± 1.15, median 5.0) on a seven-point scale, representing a meaningful 1.47-point reduction with VR technology (Fig. 3). Confidence patterns between VR and CSI showed a moderate but non-significant correlation across cases (*r* = 0.474, *p* = 0.119), suggesting that while some case-specific factors affecting confidence were shared between modalities, others were visualization-method dependent.

### Expert comparison analysis

When benchmarked against an expert reference standard, the VR group achieved 79.7% overall diagnostic accuracy compared to 84.7% for CSI, representing a 5.0 percentage point advantage for CSI.

Resectability determination showed the most pronounced divergence (VR 58.3% ± 11.8 vs. CSI 83.3% ± 10.5), a clinically significant 25.0 percentage point deficit for VR. Individual VR participants ranged from 41.7% to 75.0% accuracy, while CSI participants achieved 66.7% to 91.7%.

Vessel contact assessments revealed consistent CSI superiority across most parameters. Arterial contact accuracy favored CSI for splenic artery (VR 88.3% vs. CSI 98.3%), coeliac trunk (85.0% vs. 93.3%), and common hepatic artery (76.7% vs. 86.7%), while showing equivalent performance for aorta, superior mesenteric artery, and proper hepatic artery (both groups 90–100%). Venous assessments similarly favored CSI (portal vein: VR 63.3% vs CSI 71.7%; superior mesenteric vein: VR 71.7% vs. CSI 78.3%). The gastroduodenal artery showed the lowest accuracy in both groups (VR 55.0%, CSI 65.0%).

Vascular stenosis assessments were equivalent between modalities (both groups ~ 90–93%), while VR demonstrated superior performance for arterial variation classification (78.3% ± 8.5 vs. 71.7% ± 12.5) but inferior tumor position localization (78.3% ± 11.3 vs. 85.0% ± 6.2).

Confidence-accuracy calibration was poor in both groups but worse with VR. The correlation between confidence and accuracy was weak for VR (*r* = 0.186) and slightly better for CSI (*r* = 0.244).

### Observations and participant feedback

The VR software presented significant usability challenges. Renderings appeared at a fixed, overly distant position in coronal orientation, requiring manual repositioning and rotation to the preferred axial view for each case. The transfer function interface was too complex for practical use, preventing participants from adjusting window settings, resulting in overly bright renderings with poor calcification visibility. Despite instruction, participants struggled with the VR interface throughout. Verbal feedback emphasized dissatisfaction with default positioning and coronal orientation, which proved disorienting. Additionally, participants often dismissed anterior gastroduodenal artery (AGD) contact assessment as clinically unimportant since the AGD is typically resected regardless of tumor involvement. Two participants reported mild cybersickness but completed all assessments without discontinuation.

## Discussion

This study evaluated the impact of VR-based resectability assessment for pancreatic carcinoma by experienced hepatopancreatobiliary surgeons. The results reveal significant practical challenges that warrant careful consideration of VR before adoption in clinical practice. While both CSI and VR achieved comparable temporal efficiency for assessment tasks, the VR cohort demonstrated substantially reduced inter-rater agreement, most critically for resectability determination -the paramount clinical endpoint.

The CSI group achieved substantial inter-rater agreement for resectability assessment (κ = 0.609), exceeding the fair-to-moderate agreement typically reported in major multicenter studies (κ = 0.282–0.555).(7,8,11) This superior CSI performance likely reflects the participants' specialized expertise as hepatopancreatobiliary surgeons actively practicing at high-volume centers. The surgery-specific nature of the assessment questions demanded particular clinical background and experience. Surgical research on the impact of VR demonstrates that junior residents and medical students exhibit enhanced skill acquisition when using extended reality (XR)-based training modalities compared to traditional instructional methods (Katz et al., 2024). Junior surgical residents and medical students demonstrate significantly enhanced decision quality when using VR volumetric rendering compared to CSI slice interpretation, with studies showing improved diagnostic accuracy, faster task completion, and better spatial understanding of complex anatomical structures (Lin et al., 2019; Greuter et al., 2021). Furthermore, performance on extended reality (XR) platforms correlates positively with younger age and prior experience with video games and XR technologies (Katz et al., 2024). Consequently, it would be reasonable to expect that younger, less experienced surgeons might demonstrate superior performance on VR platforms. However, in our study the VR group's performance (κ = 0.127) represents a concerning deterioration, falling substantially below established literature benchmarks. Using Landis and Koch's widely-adopted benchmarks for kappa interpretation, the difference between VR (κ = 0.127, 'slight') and CSI (κ = 0.609, 'substantial') represents a three-category shift, constituting a meaningful difference in agreement between modalities (Landis and Koch 1977). This finding is particularly notable given that the same specialized population achieved substantial agreement with CSI, suggesting that current VR implementation may introduce systematic barriers to accurate assessment.

The literature consistently identifies the borderline resectable category as the primary source of diagnostic discord. Giannone et al., (2021) reported zero cases achieving unanimous agreement for borderline resectable classification among 22 observers, with "major disagreement"- where identical imaging received all three possible resectability classifications—occurring in 36.2% of cases (Giannone et al., 2021). Our preliminary findings suggest that VR technology, as currently implemented, may expand this problematic diagnostic gray zone rather than clarify it, potentially converting assessments that achieve substantial agreement with CSI into sources of clinical disagreement. Because vascular contact grading and NCCN resectability class directly guide the choice between upfront surgery, neoadjuvant therapy, and non-operative management, even modest inaccuracies in individual arterial and venous involvement assessments – particularly in borderline cases – can translate into clinically meaningful differences in resectability classification and patient management.

Several factors may contribute to this performance degradation. Crucially, participants had interpreted orders of magnitude more cases with CSI than VR throughout their careers, suggesting that observed reliability differences may reflect experience disparity and novelty-related confounding rather than intrinsic modality limitations. This interpretation is supported by diminished confidence levels in VR users and weak correlation between VR and CSI confidence patterns (*r* = 0.474, *p* = 0.119), indicating that clinicians process spatial information differently in immersive environments. Observations revealed clinicians struggling with spatial orientation and attempting to recreate familiar viewing planes rather than leveraging VR's three-dimensional advantages.

Technical limitations in our implementation—including fixed default orientation, spawn distance issues, and complex transfer function controls—represent systematic barriers beyond individual adaptation. The single-transfer-function approach failed to capitalize on VR's volumetric strengths while increasing cognitive load. Notably, the VR group reported orientation difficulties and increased uncertainty yet completed assessments in similar time. This suggests participants could perceive anatomical structures but found representations insufficiently clear for confident interpretation, raising questions about why uncertain users did not spend more examination time. These findings emphasize that diagnostic performance depends on specific VR visualization strategies rather than the technology itself.

**Fig. 4:**
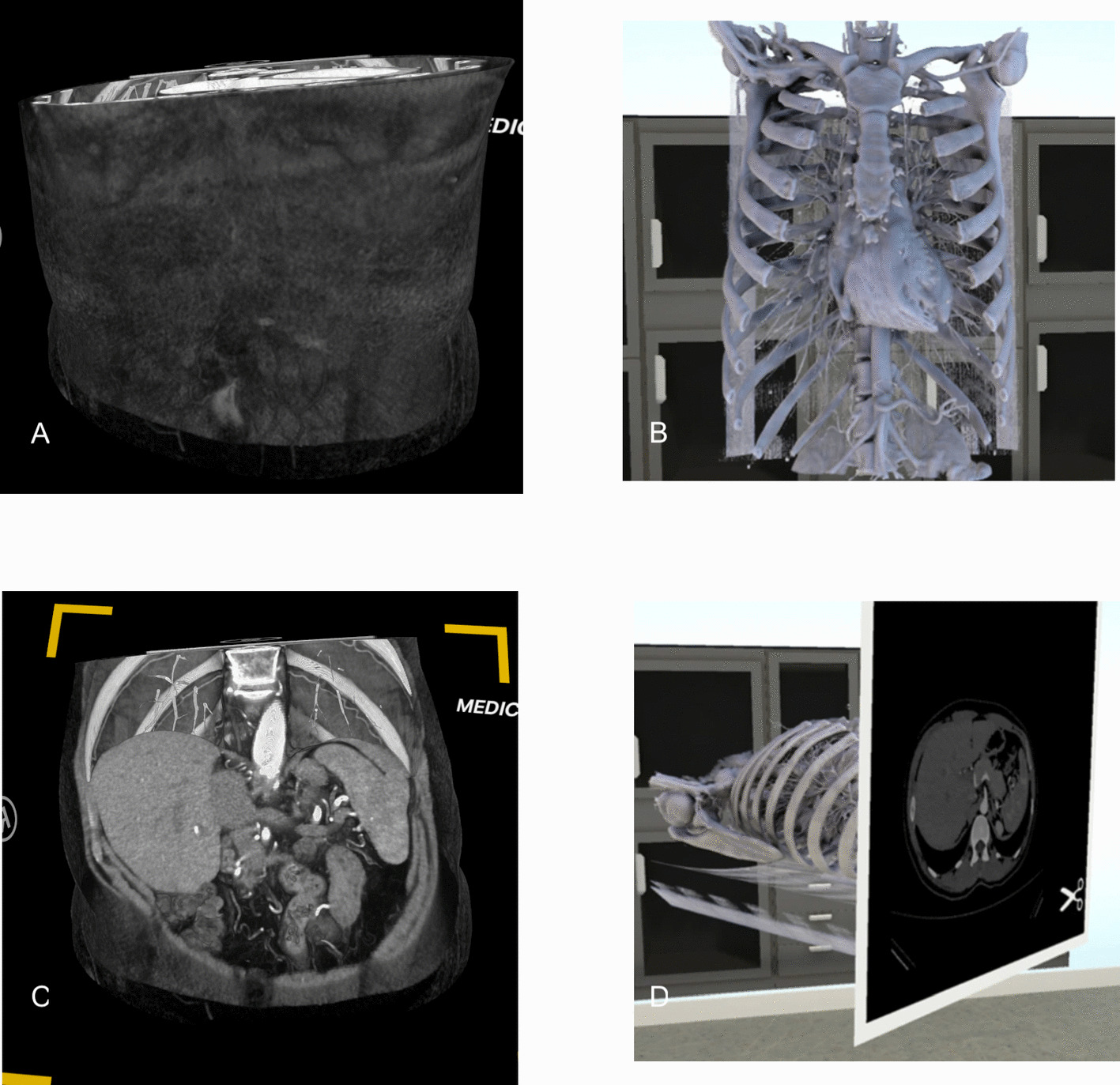
Comparison of visualization approaches: volumetric rendering versus hybrid vascular-skeletal display. Representative images comparing two distinct VR implementation strategies for pancreatic tumor assessment. Left panels show the volumetric rendering approach used in this study: coronal view (**A**) and axial view (**C**) displaying abdominal CT with minimal opacity adjustments approximating CSI abdominal windowing, maintaining visualization of all soft tissue structures including pancreas, liver, and bowel. Right panels (**B**, **D**) demonstrate the hybrid approach described by Kunz et al., displaying exclusively isolated osseous structures and contrast-enhanced vessels with integrated cross-sectional cutting planes. Original image cropped and adapted from Kunz JM, Maloca P, Allemann A, et al. Assessment of resectability of pancreatic cancer using novel immersive high-performance virtual reality rendering of abdominal computed tomography and magnetic resonance imaging. Int J Comput Assist Radiol Surg. 2024;19:1677–1687. Licensed under CC BY 4.0 (http://creativecommons.org/licenses/by/4.0/). Image cropped and combined with author's original material. CT indicates computed tomography; VR, virtual reality

Comparison with recent literature illuminates the importance of visualization methodology. Kunz et al. (2024) reported substantial inter-rater agreement for VR assessments (κ = 0.7, compared to κ = 0.04 for CSI) using a fundamentally different VR approach that displayed exclusively osseous structures and contrast-enhanced vessels, creating a combined skeletal and vascular system model (Kunz et al., 2024). While direct comparison of kappa values is limited by methodological differences – including study design (parallel randomization versus sequential assessment), reference standards (single expert versus MDT (Multidisciplinary Team) consensus), and participant composition – the contrasting outcomes highlight the importance of visualization strategy. Their interactive methodology was particularly sophisticated: when users applied cutting planes, cross-sectional slices were generated, removing model portions positioned toward the user or within the sectioned area. These slices maintained complete opacity without transparency effects, providing clear anatomical comprehension and additional orientational anchoring beyond the silhouette-based visualization achieved through our approach (Fig. 4 for comparison). This technique facilitated superior understanding of arterial variations and vascular anatomy, allowing users to rapidly access the vascular system, identify anatomical variants, and determine approximate organ positioning relative to the vascular architecture. Their implementation integrated VR advantages while preserving familiar radiological viewing conventions—a design balance we deliberately excluded to create a more controlled and equitable comparison between the VR and CSI. This methodological difference likely explains the divergent outcomes between studies and underscores that VR success depends not merely on three-dimensional display, but on thoughtful integration of interaction paradigms that enhance rather than complicate diagnostic workflows.

Based on these findings, we propose a progressive disclosure approach for future VR implementations: Initial rendering should focus on osseous structures and contrast-enhanced vessels to provide immediate anatomical context and facilitate rapid vascular assessment. Subsequently, hybrid visualization combining the initial volumetric rendering with real-time cross-sectional cutting planes would preserve radiological familiarity while leveraging VR's navigational advantages. Task-specific protocols optimized for tumor-vessel interface assessment, metastatic evaluation, or anatomical variant identification should match visual representations to cognitive demands. As clinicians gain experience with immersive visualization, entirely novel assessment paradigms may emerge that fully exploit VR's spatial advantages without relying on CSI viewing conventions.

This study has several limitations. First, the small sample size (*n* = 10 surgeons, 12 cases) limits statistical power, reduces the robustness of kappa estimates, and constrains generalizability; accordingly, these findings should be interpreted as preliminary and hypothesis-generating rather than definitive. Second, the use of a single expert radiologist as reference standard introduces potential bias and does not capture inter-expert variability in resectability assessment; future studies should employ panel consensus among multiple experts.

Despite stratified randomization by experience level, residual imbalances persisted between groups (VR group had greater surgical experience and universal prior VR exposure), which may have confounded performance differences. The brief instructional period may have been insufficient for complex diagnostic task adaptation in VR environments. Additionally, our use of one specific VR platform limits broader applicability. Importantly, VR's usability challenges, the novelty of immersive visualization, and participants' learning curves represent potential confounders that may have influenced rater behavior independent of the technology's intrinsic diagnostic capability; disentangling these effects from true modality differences requires longitudinal studies with extended training protocols. Furthermore, this study assessed inter-rater agreement and diagnostic accuracy as surrogate endpoints; improved agreement does not automatically translate to better surgical outcomes, and the clinical impact on patient outcomes requires separate prospective evaluation. Future studies should evaluate larger cohorts with younger participants, extended training periods, and multiple VR visualization strategies to establish optimal implementation approaches.

## Conclusions

Our preliminary findings suggest that VR technology's clinical utility may depend on visualization strategy rather than inherent technological limitations. While our specific implementation resulted in reduced diagnostic accuracy, Kunz et al.'s (2024) reported performance (κ = 0.7 versus CSI's κ = 0.04), though not directly comparable due to differing methodologies, suggests that appropriately designed VR visualizations may enhance interrater agreement (Kunz et al., 2024). The key distinction lies in visualization methodology: successful implementations combine volumetric advantages with familiar radiological conventions through hybrid approaches incorporating cross-sectional viewing and selective tissue visualization. Future VR development should prioritize these evidence-based design principles to realize the technology's potential for improving spatial understanding while maintaining diagnostic reliability essential for surgical decision-making.

## Supplementary Information

Supplementary Material 1.

Supplementary Material 2.

## Data Availability

The datasets generated and analysed during the current study are included in this published article as Supplementary Material 2. Participant age and years of surgical experience were removed from the published dataset to ensure anonymity.

## Abbreviations

2D: Two-dimensional
3D: Three-dimensional
AGD: Anterior Gastroduodenal Artery
CE: Conformité Européenne
CSI: Cross-Sectional Imaging
CT: Computed Tomography
DFG: Deutsche Forschungsgemeinschaft
DICOM: Digital Imaging and Communications in Medicine
FDA: Food and Drug Administration
IQR: Interquartile Range
MDT: Multidisciplinary Team
NCCN: National Comprehensive Cancer Network
PDAC: Pancreatic Ductal Adenocarcinoma
SMA: Superior Mesenteric Artery
VR: Virtual Reality
XR: Extended Reality
